# Supplementary material for: Transcriptome and Physiological Analyses of a Navel Orange Mutant with Improved Drought Tolerance and Water Use Efficiency Caused by Increases of Cuticular Wax Accumulation and ROS Scavenging Capacity
Source: Int J Mol Sci. 2022 May 18;23(10):5660. doi: 10.3390/ijms23105660 (PMC9145189; doi:10.3390/ijms23105660)
Supplement: Supplementary file 1 [file ijms-23-05660-s001.zip › Table S10.pdf]

Table S10. Primers of differentially expressed genes (DEGs) used for qRT-PCR analysis

Gene name	Accession number	Primer sequence (5'-3')
CsCER3-LIKE	Cs4g02580	CTACTGGCTTAGGGGCAGGTTG<br>TTTGCTTATTGATTCCCGTTTG
CsFAR1-LIKE	Cs8g15290	GATTTGATACCAGCGGACAT<br>AACTCGTGGATTTGGAAGAA
CsFAR3-LIKE	Cs5g15350	TACCCGTTGATATGGTGGTG<br>CAATGGGCTTTCCTTGTTTA
CsFAR4-LIKE	Cs5g15345	GGGACGATAAGTCTGCTAAA<br>ATCACCAGGCACAGCAACTA
CsNSDHL-LIKE	BGI_novel_G000534	GCTGTGGTCCTGTGGAGTGA<br>ACGGAGCTATGCAATGTTTG
CsChDI-LIKE	orange1.1t04155	ACTGTCATCGGTTTACCTTGC<br>ATCCGTAGATTCCAGCTTGTG
CsABCG11-LIKE	orange1.1t01993	TGGCAACTGCAAATGGGATA<br>GTCTTCTTGTGGAGGTGTAG
CsABCG21-LIKE	Cs7g12620	CATTGGTTATGTGCCTGGTT<br>TCGTGGTGTTCTAATCTCCC
CsMEKK1-LIKE	Cs5g22020	AAGATCCTCGAATGCAAGAG<br>CACAAGAATCAAAGCCAGAG
CsSOD1-LIKE	orange1.1t05755	ACCGTGGAAGGAAACATCTC<br>ACCGTCAACATCAACAGTGA
CsPRX5-LIKE	orange1.1t01747	TTTCCGTAGACGAGATGGTGA<br>GAAGGGTCTTGTGGGTGAGTT
CsPRX10-LIKE	Cs5g34200	TCTCAAGCTGCACTATTCCC<br>AAGCATCCCAAACACCATTC
CsERF4-LIKE	Cs1g07950	CACCTGAAGTGGCTTGAGTTT<br>GTGGAGTAACCGTCCTAGATT
CsERF9-LIKE	Cs2g23660	ATGGACGGATAGTGATGAGCC<br>TGAACTTGGACGGTTGGGTAC
CsMYB62-LIKE	Cs7g26930	AGAGGCAATCTCACTCCACA<br>CGTGCTATTTGCGTCTATCT
CsZAT10-LIKE1	Cs3g15900	TTCCTTCTCGTCCGTCTTCG<br>TTCCAGTGCCATCTCCGTTA
CsZAT10-LIKE2	Cs8g04280	GGACCCACGAGTGCTCTATTT<br>TGCTGCTGCTGTTGTTGTTGT
CsNAC22-LIKE	Cs5g29650	GCCCACAATTTATCTACGAGA<br>ATTACGATAGGAACCAGGAGC
CsWRKY27-LIKE	Cs9g19070	GCGTAAATACGGGCAGAAAC<br>TGTGAGTTGGACGAGGGTGT
CsWRKY29-LIKE	Cs5g03010	ACTAACCAGCACATCCATTT<br>GTCACCCTTTGCACTACTCT
β-actin		ACTCATCGTACTCAGCCTTTG<br>TGCACCCTGTTCTTCTTACTG
